# Design and Synthesis of Novel 1,3-Thiazole and 2-Hydrazinyl-1,3-Thiazole Derivatives as Anti-*Candida* Agents: In Vitro Antifungal Screening, Molecular Docking Study, and Spectroscopic Investigation of their Binding Interaction with Bovine Serum Albumin

**DOI:** 10.3390/molecules24193435

**Published:** 2019-09-21

**Authors:** Andreea-Iulia Pricopie, Ioana Ionuț, Gabriel Marc, Anca-Maria Arseniu, Laurian Vlase, Adriana Grozav, Luiza Ioana Găină, Dan C. Vodnar, Adrian Pîrnău, Brîndușa Tiperciuc, Ovidiu Oniga

**Affiliations:** 1 Department of Pharmaceutical Chemistry, “Iuliu Hațieganu” University of Medicine and Pharmacy, 41 Victor Babeș Street, 400012 Cluj-Napoca, Romania; pricopie.andreea@umfcluj.ro (A.-I.P.); borcea.anca@umfcluj.ro (A.-M.A.); brandu32@yahoo.com (B.T.); onigao65@yahoo.com (O.O.); 2 Preclinic Department, Pharmacy Specialization, Faculty of Medicine, Lucian Blaga University of Sibiu, 2A Lucian Blaga Street, 550169 Sibiu, Romania; 3 Department of Pharmaceutical Technology and Biopharmaceutics, “Iuliu Hațieganu” University of Medicine and Pharmacy, 41 Victor Babeș Street, 400012 Cluj-Napoca, Romania; laurian.vlase@umfcluj.ro; 4 Department of Organic Chemistry, “Iuliu Hațieganu” University of Medicine and Pharmacy, 41 Victor Babeș Street, 400012 Cluj-Napoca, Romania; adriana.ignat@umfcluj.ro; 5 Research Center on Fundamental and Applied Heterochemistry, Faculty of Chemistry and Chemical Engineering, “Babeş-Bolyai” University, 11 Arany Janos Street, 400028 Cluj-Napoca, Romania; lusi@chem.ubbcluj.ro; 6 Department of Food Science and Technology, University of Agricultural Sciences and Veterinary Medicine, 3-5 Mănăștur Street, 400372 Cluj-Napoca, Romania; dan.vodnar@usamvcluj.ro; 7 National Institute for Research and Development of Isotopic and Molecular Technologies, 67-103 Donath Street, 400293 Cluj-Napoca, Romania; adrian.pirnau@itim-cj.ro

**Keywords:** 1,3-thiazole, anti-*Candida*, molecular docking, bovine serum albumin, fluorescence quenching

## Abstract

In the context of there being a limited number of clinically approved drugs for the treatment of *Candida* sp.-based infections, along with the rapid development of resistance to the existing antifungals, two novel series of 4-phenyl-1,3-thiazole and 2-hydrazinyl-4-phenyl-1,3-thiazole derivatives were synthesized and tested in vitro for their anti-*Candida* potential. Two compounds (**7a** and **7e**) showed promising inhibitory activity against the pathogenic *C. albicans* strain, exhibiting substantially lower MIC values (7.81 μg/mL and 3.9 μg/mL, respectively) as compared with the reference drug fluconazole (15.62 μg/mL). Their anti-*Candida* activity is also supported by molecular docking studies, using the fungal lanosterol C14α-demethylase as the target enzyme. The interaction of the most biologically active synthesized compound **7e** with bovine serum albumin was investigated through fluorescence spectroscopy, and the obtained data suggested that this molecule might efficiently bind carrier proteins in vivo in order to reach the target site.

## 1. Introduction

The increasing incidence of *Candida* sp.-based infections poses a direct threat to the healthcare system, being associated with high mortality rates and expensive medical costs for governments and hospitalized patients [1]. Currently, only five classes of antifungal drugs are available for the treatment of superficial and systemic fungal infections. The most widely used, the azole compounds, are directed against ergosterol biosynthesis through the inhibition of lanosterol-C14α-demethylase (CYP51). The resulted ergosterol depletion and the methylated sterols accumulation lead to alterations in the integrity and the functioning of the fungal cell membrane, thus inhibiting fungal cell replication and enhancing susceptibility to host defense mechanisms [2]. The effectiveness of these marketed drugs is significantly reduced by the development of robust resistance mechanisms in medically important fungal strains, such as drug target overexpression, the up-regulation of drug efflux pumps, and activation of the stress response [3]. Furthermore, the development of novel chemotherapeutic agents, with a distinct mechanism of action, is hindered by the phylogenetic relatedness between fungi and humans, and is responsible for the reduced number of differential targets to be exploited [4]. In this setting, the development of novel chemical entities that are endowed with lanosterol C14α-demethylase enzyme inhibitory potential and are less susceptible to the above-mentioned fungal adaptation mechanisms is a priority.

The identification of drug-like chemical features and theoretical ligand structures as biologically active hits or leads is easily achievable nowadays due to the availability of high resolution structural analysis techniques such as X-ray crystallography and in silico drug design tools. Both structure-based molecular docking programs and ligand-based molecular modeling studies can be employed for this purpose [5]. 

Nitrogen-containing heterocycles have received particular interest in recently reported research studies due to their valuable biological activities [6]. In particular, 1,3-thiazole scaffold is a versatile building block for lead generation, and is readily accessible for subsequent lead optimization through chemical derivatization. The antifungal activity against clinically relevant *Candida* sp. strains was described for a large number of small molecules bearing thiazole scaffolds [7,8]. 

The main structural features identified in the family of 1,3-thiazole derivatives with antifungal activity by means of pharmacophore-based approaches consist of: An aromatic nitrogen fragment with an accessible lone pair, functioning as a hydrogen bond acceptor, a hydrophobic area represented by a phenyl ring with a para-lipophilic substituent, along with a second aromatic ring and a hydrogen bond donor motif [9,10]. 

**Figure 1:**
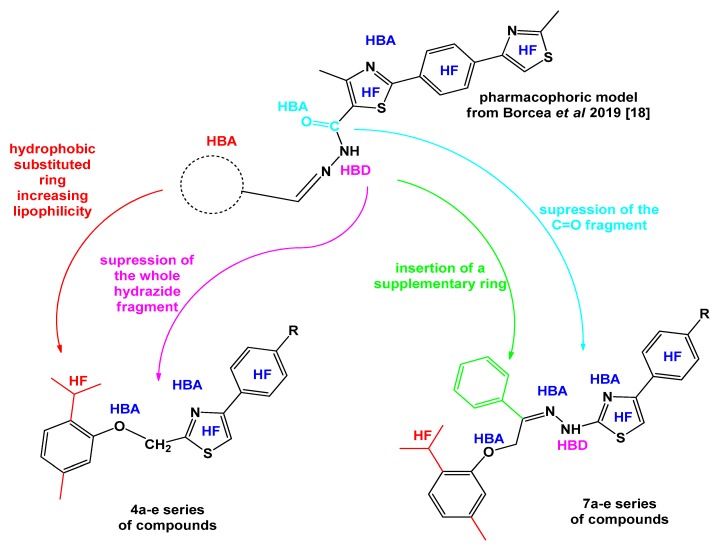
Rational design of the titled 4-substituted thiazole derivatives (HF–hydrophobic feature; HBA–hydrogen bond acceptor; HBD–hydrogen bond donor).

Prompted by this validated hypothesis, and maintaining consistency with our research group interest for thiazole-based compounds, we decided to synthesize a novel series of molecules containing this chemical feature in order to investigate their antifungal potential. On account of the recently reported data regarding the importance of a hydrazine substituent at the C_2_ position of the thiazole ring in terms of improved anti-*Candida* activity [11,12], we were encouraged to work toward the chemical development of a second series of substituted-2-hydrazinyl-1,3-thiazole derivatives (Figure 1).

The inhibitory activity of the newly synthesized compounds **4a**–**e** and **7a**–**e** was evaluated in vitro against opportunistic *Candida* sp. strains and the results obtained, which were expressed in terms of minimum inhibitory concentration (MIC) and minimum fungicidal concentration (MFC), were compared to those of fluconazole, which was used as a reference drug. In order to confirm their lanosterol-C14α-demethylase inhibitory potential, both series were subjected to a molecular docking study. Additionally, their drug-like properties were assessed through an in silico absorption, distribution, metabolism, excretion and toxicity (ADMET) prediction. It has been shown that the pharmacokinetic properties, as well as the stability and toxicity of a drug, are actively influenced by drug–protein interactions in the blood stream [13]. Human serum albumin (HSA) is an abundant plasma protein that binds a wide range of drugs and metabolites, so the investigation of binding interactions between the biologically active small molecules and serum albumin is an important step for the assessment of their distribution and pharmacological activity. Bovine serum albumin (BSA) and HSA are homologous proteins, and their interactions with different drugs have been extensively studied by using both experimental and theoretical calculation methods. Due to its low cost and highly structural homology with HSA, BSA represents the serum albumin that is preferred in laboratory practice for the above-mentioned type of pharmacokinetic studies [14]. Fluorescence spectroscopy was employed for the analysis of the binding profile of the most promising of the newly synthesized compounds with BSA in terms of binding mechanism, binding constants, and the number of binding sites.

## 2. Results and Discussion

### 2.1. Chemistry

**Scheme 1:**
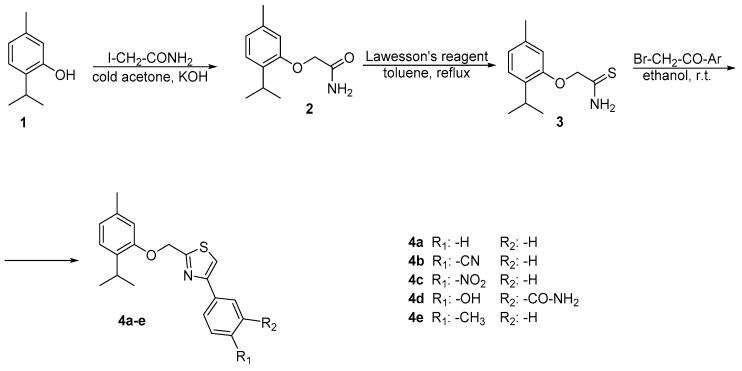
Synthetic protocol of the 4-phenyl-1,3-thiazole derivatives **4a**–**e**

The synthetic protocol employed for the chemical development of the first series of 4-phenyl-1,3-thiazole compounds (**4a**–**e**) is outlined in Scheme 1. 

The O-alkylation reaction of the phenolic hydroxyl group of thymol (**1**) with 2-iodoacetamide yielded the amide compound **2**. Next, a thionation process using Lawesson’s reagent (LR) in refluxing toluene, followed by the Hantzsch condensation of the intermediate thioamide **3** with the appropriate α-haloketones afforded the targeted 4-phenyl-1,3-thiazole derivatives.

**Scheme 2:**
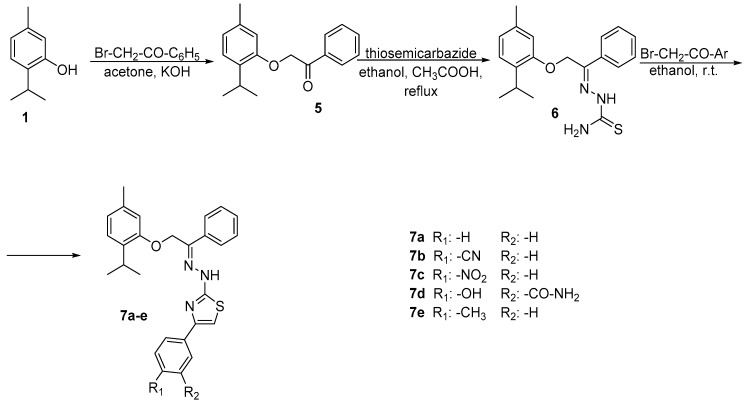
Synthetic protocol of the 2-hydrazinyl-4-phenyl-1,3-thiazole derivatives **7a**–**e**.

The second series of compounds was synthesized according to the route described in Scheme 2. By reacting thymol with 2-bromoacetophenone in alkaline media, we obtained the ketone derivative **5**. The desired 2-hydrazinyl-4-phenyl-1,3-thiazoles (**7a**–**e**) were synthesized in a two-step process, by classical condensation of the carbonyl group with thiosemicarbazide in refluxing ethanol, in the presence of glacial acetic acid as catalyst, followed by heterocyclization with the corresponding α-halocarbonyl compounds.

The reaction progress was periodically monitored by thin-layer chromatography (TLC). The structures of the newly synthesized compounds were assigned by elemental analysis and spectral data: Fourier transform infrared spectroscopy (FT-IR), electrospray ionization-mass spectrometry (ESI-MS), hydrogen nuclear magnetic resonance (^1^H-NMR) and carbon nuclear magnetic resonance (^13^C-NMR). The results of the C, H, N, and S quantitative elemental analysis of the titled thiazole derivatives were consistent with the calculated values, within ±0.4% of the theoretical values. The recorded molecular ion peaks were consistent with their molecular formulas. Concerning compounds **4a**–**e**, the thionation of the intermediate amide **2** was confirmed by the FT-IR spectral data through the disappearance of the high-intensity signal corresponding to the carbonyl group stretching vibration at 1698 cm^−1^. Subsequent cyclization to the desired 4-phenyl-1,3-thiazoles is supported by the appearance in their IR spectra of a sharp medium intensity signal at 3180–3100 cm^−1^, corresponding to the thiazole-methine group stretching vibration, as well as by MS and NMR analysis. Spectroscopic data related to the methine located at the C_5_ position of the thiazole ring are highly specific for this class of compounds [15]. Regarding the newly synthesized 4-phenyl-1,3-thiazole derivatives, the presence of a singlet at around 7.62–8.55 ppm in the ^1^H-NMR spectra, along with the ^13^C-NMR corresponding signal at about 110–115 ppm, confirmed their proposed structure.

The structure of the thiosemicarbazone intermediate **6** was confirmed by the characteristic signal in the ^13^C-NMR spectra, at 145.3 ppm, belonging to the tertiary carbon atom involved in the hydrazone bridge (C=N), as well as the two broad signals in the ^1^H-NMR spectra, at 10.80 ppm and 8.07 ppm, respectively, corresponding to the –NH and –NH_2_ protons. The successful accomplishment of Hantzsch condensation is also supported by ^1^H-NMR through the disappearance of the NH_2_-related signals together with the appearance of thiazole-methine proton specific singlet, at 8.19–8.54 ppm, and additional aromatic protons, according to the specific structure of **7a**–**e** derivatives.

### 2.2. Anti-Candida Activity Assay

The antifungal potential of the newly synthesized compounds was evaluated in vitro against three human pathogenic *Candida* strains. The broth microdilution method was employed for the determination of MIC and MFC values. Stock solutions (1 mg/mL) were prepared by dissolving the tested compounds and the reference antifungal drug, fluconazole, in sterile DMSO. The obtained values are presented in Table 1 and Table 2.

The newly synthesized compounds exhibited moderate to good anti-*Candida* activity, according to the obtained MIC and MFC values. The best inhibitory activity was exerted against *C. albicans* strain ATCC 10231, the non-albicans species being less susceptible to almost all of the tested molecules.

The 2-hydrazinyl-4-phenyl-1,3-thiazole derivatives (**7a**–**e**) proved to be superior to those lacking the C_2_-hydrazone linkage (**4a**–**e**) in terms of antifungal potency. This superiority may be explained either by the increased lipophilicity of the former, which is supported by the calculated logP values, and correlates with an increased ability to penetrate the fungal cell membrane. Compounds **7a** and **7e** were the most promising, exhibiting substantially lower MIC values (7.81 μg/mL and 3.9 μg/mL, respectively) as compared with the reference drug fluconazole (15.62 μg/mL). 

Some structure-dependent differences in activity could be noticed in the second series of compounds (**7a**–**e**), which was related to the para-substitution of the C_4_-phenyl ring. It seems that the presence of a lipophilic, electron-donating substituent (–CH_3_) is correlated with an increased inhibitory activity on both *albicans* and non-*albicans* Candida strains, as compared with hydrophilic, polar substituents (–CN, –NO_2_, –OH). These findings are in accordance with the data reported in the literature regarding the antifungal potential of hydrazinyl-thiazoles with hydrophobic features and are also supported by the results obtained from the in silico antifungal screening [9,11].

The determination of MFC confirmed the previously obtained MIC values. The MFC/MIC ratio values for all the tested compounds were equal to two, suggesting that they may exert a fungicidal effect [16].

### 2.3. Molecular Docking

Docking simulation is a widely used structure-based drug design (SBDD) method because of its ability to predict the affinity of the ligands toward the active site of the biological targets through the prediction of the interaction energy associated with each of the potential binding conformations [17]. The rational design and development of antifungals is particularly challenging, as approximately 80% of the antifungal targets reported in the literature turned out to be false positive, with little potential to develop target-based inhibitors. Another aspect worth mentioning is the potential lead-like molecules’ lack of selectivity toward fungal enzymatic machinery. Lanosterol-C14α-demethylase is a validated target in the fungal cell membrane that is not present in the host cell membrane, which explains the growing interest for the discovery of novel compounds that are capable of inhibiting it [2].

In order to evaluate the binding affinity of the newly synthesized compounds toward the catalytic site of the target enzyme, a molecular docking study was carried out. For each compound, the conformation with the best binding affinity, expressed as the highest variation of Gibbs-free energy (∆G) of the complex with the target CYP51, and the computed inhibition constant (Ki) are presented in Table 3, together with the conformational analysis of the cluster containing the top binding conformations. 

The results of the molecular docking found poses with favorable binding energies toward *C. albicans* lanosterol C14α-demethylase for all the tested molecules. The differences observed, in terms of interaction energy (ΔG), might be correlated with the C_2_-substitution of the thiazole ring. 

According to the obtained data, the presence of the hydrazone bridge and the additional aromatic phenyl in the second series of 2-hydrazinyl-4-phenyl-1,3-thiazoles (**7a**–**e**) improved their affinity for the target fungal enzyme, as compared with the first series of 4-phenyl-1,3-thiazole derivatives (**4a**–**e**). An aspect worth mentioning here is the higher flexibility of the compounds **7a**–**e**, as compared to **4a**–**e**, which lack the C_2_-substituent, as outlined by the increased number of clustered conformations with improved binding affinity to the target enzyme. Moreover, the hydrogen-bonding capacity of *N*_1_-hydrazine nitrogen allows additional interaction with the target lanosterol-C14α-demethylase. 

For the compounds **4a**–**e**, some important structural rigid elements, such as aromatic nuclei or the sp^2^ hybrid imine carbon, are responsible for the heterogeneity of their binding mode. These molecules have an important number of clusters containing residual conformations, which don’t match the main cluster. The influence of structural flexibility on ΔG is also supported by the larger dispersion of Gibbs free energy values and the large spatial dispersion of the predicted poses in the case of the second series of compounds (**7a**–**e)**.

The observed differences in terms of ΔG for compounds from both series could be attributed to the lipophilicity and mesomeric effects of the para-substituent of C_4_-phenyl ring. It could be noticed that the presence of a lipophilic, electron-donating substituent (–CH_3_) in compounds **4e** and **7e** is associated with improved binding energy, as compared with the hydrophilic, electron withdrawing functional groups (–CN, –NO_2_), given the hydrophobic environment (Phe233, Val510 amino acid residues) of the phenyl-thiazole fragment. The two distinct areas with different polarity identified in the binding regions from the access channel to the catalytic site of the lanosterol C14α-demethylase were thoroughly described in a previous study reported by our research group [18]. 

**Figure 2:**
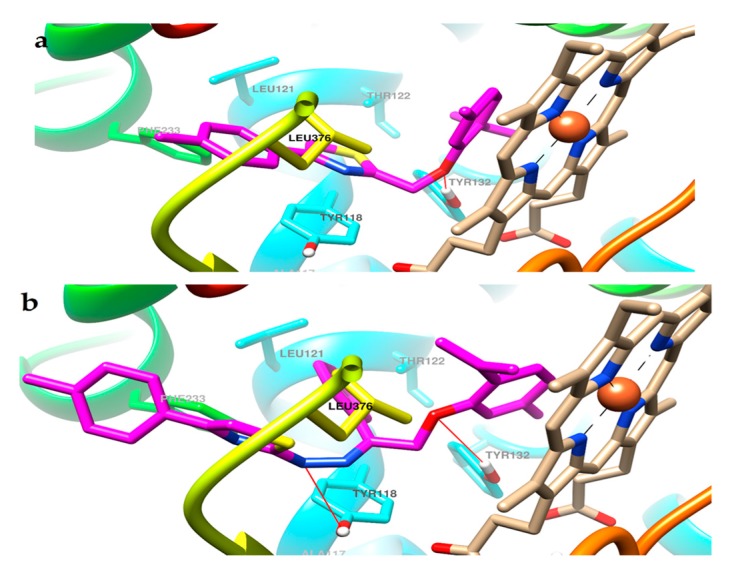
The top binding conformation of compounds **4e** (subsection (**a**)) and **7e** (subsection (**b**)). C atoms of the docked compounds to the active site of fungal lanosterol C14α-demethylase are depicted in magenta. Unnecessary protein fragments in the background and foreground were removed for clarity.

A comparative analysis of the linking mode of the most active compound **7e** with its structural analog **4e** was made. In Figure 2, significant differences can be observed between the two. Both compounds **4e** and **7e** enter the depth of the enzyme’s catalytic pocket along the access channel, with the thymol fragment parallel to the active site, favoring a п-cation interaction. The 2-phenyl-thiazole fragment of both compounds **4e** and **7e** fits well between the hydrophobic Leu376 and Phe233. On the other side, the Leu121, Phe233, and Thr122 amino acid residues create another lipophilic subpocket into the nonpolar zone of the binding pocket, which fits the supplementary phenyl ring of the compound **7e**. Moreover, for the compound **7e**, the presence of the hydrazine bridge allows additional interactions with the polar area of the access channel. The electron lone pair of the *N*_1_-hydrazine nitrogen allows the interaction of the compound with the polar Tyr118 residues through a hydrogen bond. For both compounds **4e** and **7e**, a second polar interaction with the Tyr132 residue is endowed by the etheric oxygen, which also acts as a hydrogen bond acceptor.

According to the data obtained from the molecular docking study, the newly synthesized compounds might act as noncompetitive inhibitors of the fungal lanosterol C14α-demethylase. The induced enzyme inhibition is not related to the covalent coordination of the heme Fe^2+^, as in the case of classical antifungal azoles, but they compete with the physiological substrate of the enzyme for access to the active site. This mechanism of action has been reported in the literature as being associated with a reduced toxicity and resistance of fungal pathogens to the clinically approved azole drugs [19,20]. Further studies on the fungal sterols’ biosynthetic pathway, involving ergosterol extraction and quantitation assay, are required in order to confirm the inhibitory effect of the tested thiazole derivatives against the target enzyme.

### 2.4. ADMET Profiling

In silico ADMET prediction studies are a central component of pharmaceutical research and drug design, as it provides helpful guidance in the early evaluation of the in vivo efficiency and safety of a drug. The magnitude of the biological activity of an active molecule, in terms of specific interactions with the molecular target, along with the drug-related side effects, are strongly influenced by its pharmacokinetic properties [21]. Integrated ADMET prediction platforms allow an early research stage determination of the molecular descriptors, which predicts the suitability of a small molecule for oral administration, thus reducing the number of synthesis–evaluation cycles and the more expensive late-stage failures [22].

Drug-likeness descriptors of the newly synthesized compounds, according to the Lipinski Rule of Five [23], are presented in Table 4.

According to the Lipinski Rule of Five, a newly synthesized small molecule is more likely to be an orally active drug if it has no more than one violation of the following criteria: a molecular weight under 500 Da, an octanol–water partition coefficient (the Moriguchi logarithm of the compound partition coefficient, or mLogP) lower than five, less than 10 nitrogen and oxygen atoms in the molecule, and no more than five potential hydrogen donors [24]. As it can be observed in Table 4, the newly synthesized 4-phenyl-1,3-thiazole **4a**–**e** and 2-hydrazinyl-4-phenyl-1,3-thiazole derivatives **7a**–**e** meet the criteria for drug-likeness. It is worth mentioning that all the tested compounds comply with Veber’s rule regarding the influence of polar surface area and molecular flexibility on oral bioavailability [25]. 

The predicted molecular properties correlated with the pharmacokinetic profile and central nervous system (CNS) activity of the titled compounds are presented in Table 5.

Permeability through biological cell membranes, including the gastrointestinal tract barrier, proved to be predicted by a series of molecular properties, such as the partition coefficient (logP), topological polar surface area (tPSA), intrinsic water solubility, carrier proteins-mediated efflux, and molecular flexibility [26]. The cell membrane partitioning of a drug is considered a two-step process. In the first step, drug incorporation into the phospholipid bilayer is strongly dependent on its lipophilicity. In the second step, by contrast, drug delivery through the interior of the phospholipid bilayer is dependent on its hydrogen-bonding capacity and polarity. 

The dependence of gastrointestinal absorption on tPSA is assessed by Equation (1) [27]:

%GI Abs = 109 − (0.345 × tPSA)(1)

Drugs that are almost completely absorbed (>90%) after oral administration have a tPSA < 60 Å, while a tPSA > 140 Å results in unacceptably low (<10%) oral absorption [28]. 

As is can be observed in Table 4, all the newly synthesized compounds have tPSA values under the threshold of 140 Å, which might suggest a good oral bioavailability. The lower values, which are associated with a higher level of gastrointestinal absorption (%GI Abs = 69.78–91.62%), were recorded for the 4-phenyl-1,3-thiazole derivatives (**4a**–**e)**. The slightly reduced oral bioavailability (%GI Abs = 61.36–83.21%) of the 2-hydrazinyl-4-phenyl-1,3-thiazole derivatives (**7a**–**e)** may be explained by their higher polarity and increased molecular flexibility, owing to the superior number of rotatable bonds [25,28].

Classification of a drug as CNS active or inactive is based on evidence related to its blood–brain barrier (BBB) permeability, which can be evaluated based on the predicted molecular weight (MW), mLogP, HBA, HBD, and tPSA [16]. In particular, a tPSA value under 60–70 Å tends to identify CNS active compounds [23]. The vast majority of the tested compounds have no ability to penetrate the blood–brain barrier, thus presenting a low risk for CNS side effects. Only one compound, **7a**, exhibits BBB penetration, which may be explained by its proper lipophilicity and lower molecular weight [29,30]. 

Lanosterol C14α-demethylase (CYP51) belongs to the cytochrome P450 superfamily, which is a large class of hemoproteins involved in the metabolism of a wide variety of endogenic metabolism products and xenobiotics. Due to the high homology in the binding pocket of the CYP450 isoforms, the development of selective inhibitors represents an important challenge in the field of drug design [31]. The clinically approved CYP51 inhibitor fluconazole is known to be a moderate inhibitor of CYP2C9 and CYP3A4 isoenzymes and is recommended in regulatory guidance as a prototype inhibitor to assess the potential for drug–drug interactions mediated through CYP2C9 inhibition [32]. 

The ligand-induced CYP450 cross-inhibition proved to be influenced by a series of molecular descriptors, such as the molecular size and shape, hydrogen-bonding capacity, mLogP, and molecular weight [33].

In respect of the newly synthesized compounds, the obtained results showed that the majority of 2-hydrazinyl-4-phenyl-1,3-thiazole derivatives (**7a**, **7c**–**e**) exhibit no cross-inhibition of the human CYP2C9 isoenzyme, thus being less susceptible for drug–drug interactions-mediated side effects. It may be explained by their large molecular size, compared with CYP2C9 substrates, and their higher hydrogen-bonding capacity. The lower hydrophobicity and the presence of more hydrogen bond donors and acceptors as compared with the substrates may also explain the higher degree of CYP3A4 cross-inhibition potential for both series of tested molecules [32].

### 2.5. Protein-Binding Study

The antimicrobial potency of heterocyclic compounds may be hindered by their restricted access to the target site in vivo, because of the hydrophobicity of these molecules [34]. The absorption of a drug into the blood stream and its distribution to the molecular target depend largely on its interactions with carrier proteins, such as serum albumin or α_1_-acidic glycoprotein (α_1_-AGP) [35]. Human serum albumin (HSA) is the most abundant carrier protein in plasma, having a high affinity for a wide range of exogenous and endogenous ligands. HSA increases the apparent plasma solubility of hydrophobic drugs and modulates their pharmacokinetic properties, free active concentration, and toxicity [36]. The nature and magnitude of small molecules’ binding interactions with HSA represent an important predictor of their efficacy and delivery rate to the specific cellular target in vivo [37]. Therefore, the investigation of the main factors involved in this process may be useful for the rational design and development of novel derivatives with improved biological activity [38]. Given the high structural homology with HSA and its relatively low cost, bovine serum albumin (BSA) is often used in laboratory practice for the establishment of the protein-binding profile of various drugs [14]. 

On account of the intrinsic fluorescence of HSA, originating from tryptophan (Trp) residues, the measurement of fluorescence quenching is a suitable analytical method to study drug–protein interactions [39]. The fluorescent emission of serum albumin is sensitive to changes in the local environment of the Trp-134 and Trp-212 amino acid residues, which is induced by the binding of a small molecule [34]. The ligand binding induced decrease in intensity of the Trp fluorescence may occur through a static or/and dynamic quenching mechanism, and is mediated by different types of molecular interactions, such as ground state complex formation, excited state reactions, energy transfer, and collision quenching [37]. In the case of static quenching, the ligand binding to the macromolecule takes place in the ground state, and the resulting complex is nonfluorescent in nature. By contrast, in the case of dynamic quenching, the fluorophore residues interact with the quencher during the excitation [40]. 

**Figure 3:**
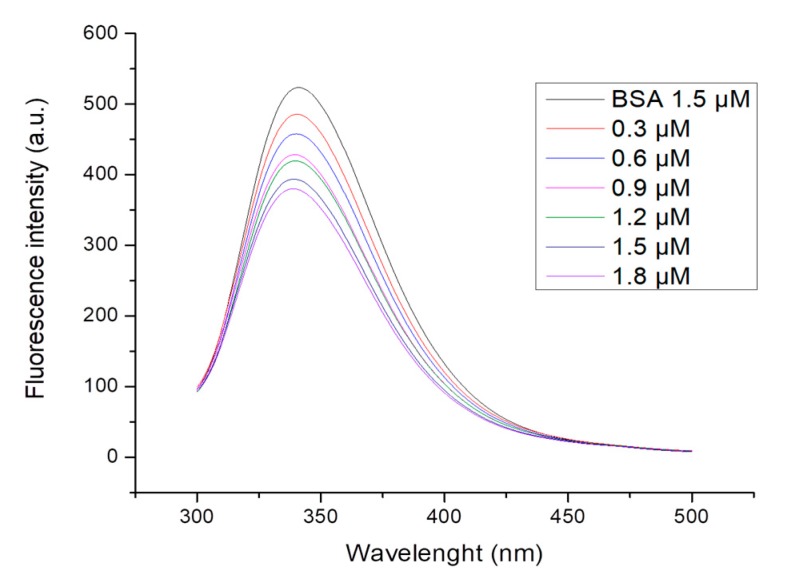
Fluorescence spectra of bovine serum albumin (BSA) (1.5 μM; λmax = 341 nm) in the presence of compound **7e** (0.3 μM; 0.6 μM; 0.9 μM; 1.2 μM; 1.5 μM 1.8 μM; λmax = 339 nm).

In order to analyze the binding profile of the most biologically active molecule of the newly synthesized thiazole derivatives, the fluorescence spectra of BSA upon the addition of an increasing concentration of compound **7e** (0.3 μM; 0.6 μM; 0.9 μM; 1.2 μM; 1.5 μM 1.8 μM) were recorded. Spectral analysis was performed at room temperature by excitation of the probes at 289 nm (λex) and an emission scan from 300 to 500 nm. On excitation at 289 nm, the emission spectrum of BSA showed an intense band at 341 nm. The obtained data demonstrated a linear decrease of BSA fluorescent emission on increasing ligand concentration, as illustrated in Figure 3. A slight hypsochromic shift of the BSA maximum emission wavelength was also observed from 341 nm to 339 nm, these suggesting a static quenching mechanism [36,41].

This blue shift indicates that the ligand binding and formation of a ground-state complex with BSA expose the fluorophore Trp residues to a more hydrophobic environment, thus changing their emission properties [41].

The magnitude of fluorescent quenching of the protein induced by the tested compound **7e** and the mechanism involved in the process were investigated using the Stern–Volmer Equation (2) [14]:

F0/F = 1 + K_SV_ × [Q] = 1 + K_q_ × τ_0_ × [Q] or (F0 − F)/F = K_SV_ × [Q] = K_q_ × τ_0_ × [Q] (2)

where F0 and F are the fluorescence intensities of BSA in the absence and presence of the quencher, respectively; K_SV_ is the Stern–Volmer quenching constant, and [Q] represents the concentration of the quencher. K_q_ is the quenching rate constant of the biomolecule, and τ_0_ is the average fluorescence lifetime of the biomolecule without the quencher, which is about 6 ns for BSA. 

**Figure 4:**
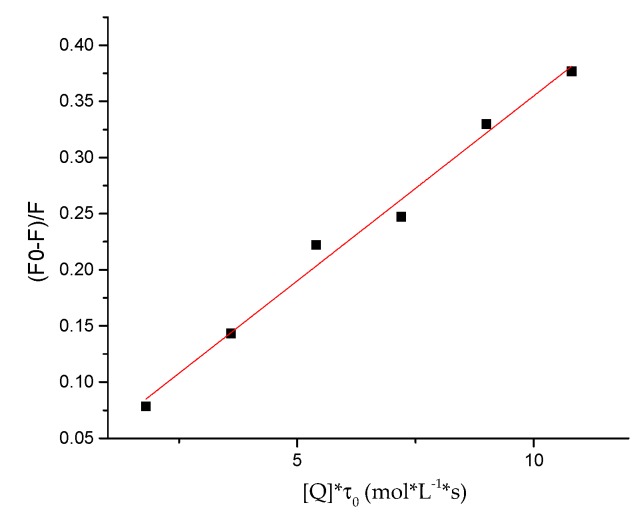
The Stern–Volmer curves for the quenching of BSA (1.5 μM) by **7e** (0.3 μM; 0.6 μM; 0.9 μM; 1.2 μM; 1.5 μM 1.8 μM) (λex = 289 nm, T = 298K).

K_SV_ and K_q_ were determined by the slope and the intercept of the linear regression plot graph of the relative emission intensity (F0 − F)/F versus τ_0_ × [Q], as shown in Figure 4. The calculated values for the compound **7e** are presented in Table 6.

K_q_ values greater than that of the maximum scatter collision quenching constant with biopolymers (2 × 10^10^ L·mol^−1^·s^−1^) confirm the static quenching of BSA induced by the newly synthesized compounds through the formation of a ground-state complex [39].

For the static quenching interaction, the number of binding sites (n) and the binding constants (K_b_) can be determined by the intercept and the slope of the regression curve, using Equation (3) [42]. The obtained results are presented in Table 7.

log[(F0 − F)/F] = log K_b_ + n log[Q] (3)

The strength of the binding interaction is assessed based on the values of K_b_: the higher the value of K_b_, the stronger the binding between the protein and the ligand [40].

The obtained n value was about 1, indicating the existence of a single binding site of compound **7e** on BSA. The k_b_ values are in the order of 10^5^, which support a strong binding interaction and a high affinity of the macromolecule for the tested ligand [43].

As reported for clinically approved azole antifungals, an improved uptake of drugs into the fungal cells and a more rapid inhibition of cellular replication may be associated with a higher affinity and binding interaction with the serum albumin [44]. In this setting, the binding profile of the tested compounds suggests that HSA may act as a drug delivery system, improving the targeting and decreasing their plasma-free concentration and toxicity. 

## 3. Materials and Methods 

### 3.1. General Information

All the chemicals and reagents used for synthesis were obtained from Alfa Aesar (Karlsruhe, Germany) and Merck (Darmstadt, Germany), and were used as supplied, without further purification. Analytical thin-layer chromatography (TLC) carried out on Merck precoated Silica Gel 60F254 sheets (Darmstadt, Germany) was employed for the monitoring of the reaction progress and confirming the purity of the newly synthesized compounds, using a mixture of ethyl acetate:n-hexane = 3:1 as the elution system and UV light (254 nm) for visualization. Melting points were determined with an electrothermal melting point meter through the open glass capillary method and are presented uncorrected. Spectral analytical methods (mass spectrometry [MS], infrared spectroscopy [IR], and nuclear magnetic resonance [NMR]) were used to confirm the structures of the synthesized compounds. IR spectra were recorded on a Jasco FT-IR 6100 spectrometer (Jasco, Easton, MD, USA), using anhydrous potassium bromide for sample preparation. MS analyses were performed in positive ionization at 70 eV, using an Agilent 1100 series and an Agilent Ion Trapp SL mass spectrometer (Agilent, Santa Clara, CA, USA). ^1^H-NMR spectra were recorded on a Bruker Advance NMR spectrometer (Karlsruhe, Germany), operating at 500 MHz, using DMSO-d_6_ as the solvent and tetramethylsilane (TMS) as the internal standard. ^13^C-NMR analyses were performed on a Bruker Advance NMR spectrometer operating at 125 MHz, in DMSO-d_6_ and using a Waltz-16 decoupling scheme, with TMS as the internal standard. Chemical shift (δ) values were reported in parts per million (ppm). Spin multiplets are given as s (singlet), d (doublet), t (triplet), and m (multiplet). For the elemental analysis, a Vario El CHNS instrument (Hanau, Germany) was employed.

### 3.2. Chemistry

#### 3.2.1. Synthesis of 2-(2-isopropyl-5-methylphenoxy)acetamide **2**

A solution of thymol (10 mmol) and potassium hydroxide (KOH) (11 mmol) in dry acetone was cooled at 0 °C in an ice–salt bath. Then, equimolar quantities of 2-iodoacetamide were slowly added, and the reaction mixture was further stirred at room temperature for 3 h. The obtained white solid mass was filtered under vacuum, washed thoroughly with a mixture of ethanol:water = 1:3, and dried. 

Yield 87%; m.p. 99.5–100.5 °C; FT-IR (KBr) ν_max_ cm^−1^: 3424-3283 (N–H str), 3055 (C-Har str), 2932 (C-Halif str), 1698 (C=O amide str), 1613 (C=N str), 1257 (C–O–C asym str), 1064 (C–O–C sym str); ^1^H-NMR (500 MHz, DMSO-d_6_, δ/ppm): 7.19 (s, 2H, –NH), 7.10 (d, *J* = 7.50, 1H, Ar–H), 6.78 (d, *J* = 7.50, 1H, Ar–H), 6.66 (s, 1H, Ar–H), 4.38 (s, 2H, O–CH_2_), 2.50-2.51 (m, 1H, Ar–CH–(CH_3_)_2_), 2.19 (s, 3H, Ar–CH_3_), 1.09 (d, *J* = 6.84, 6H, Ar–CH–(CH_3_)_2_); ^13^C-NMR (125 MHz, DMSO-d_6_, δ/ppm): 166.3 (C=O), 155.1 (C), 136.3 (C), 132.6 (C), 126.1 (CH), 122.5 (CH), 113.6 (CH), 65.3 (CH_2_), 25.9 (CH), 23.3 (2CH_3_), 21.3 (CH_3_); MS (ESI) *m*/*z*: [M + H]^+^ 208.1; anal. calculated for C_12_H_17_NO_2_ (%): C, 69.54; H, 8.27; N, 6.76; found (%): C, 69.77; H, 8.38; N, 6.71.

#### 3.2.2. Synthesis of 2-(2-isopropyl-5-methylphenoxy)ethanethioamide **3**

To a solution containing 10 mmol of compound **2** in 30 mL of anhydrous toluene, 12 mmol of Lawesson’s reagent (LR) were slowly added, under vigorous stirring. The reaction mixture was further refluxed at 150 °C, under continuous stirring, until the TLC (ethyl acetate:n-hexane = 3:1 *v*/*v*) confirmed the completion of the chemical process (3 h). The solid mass was filtered off in order to get rid of the excessive LR and insoluble impurities, and the filtrate was concentrated under reduced pressure, using a rotary evaporator. The cooled residue in a mixture of ethanol:diethylether = 1:1 was filtered under vacuum to yield the desired carbothioamide **3**. 

Yield 51%; m.p. 125 °C; FT-IR (KBr) ν_max_ cm^−1^: 3407–3229 (N–H str), 3052 (C-Har str), 2925 (C-Halif str), 1617 (C=N str), 1254 (C-O-C asym str), 1117 (C=S str), 1049 (C–O–C sym str); ^1^H-NMR (500 MHz, DMSO-d_6_, δ/ppm): 9.99 (s, 1H, –NH), 9.09 (s, 1H, –NH), 7.10 (d, *J* = 7.50, 1H, Ar–H), 6.78 (d, *J* = 7.50, 1H, Ar–H), 6.66 (s, 1H, Ar–H), 4.73 (s, 2H, O–CH_2_), 2.50-2.51 (m, 1H, Ar–CH–(CH_3_)_2_), 2.24 (s, 3H, Ar–CH_3_), 1.15 (d, *J* = 6.85, 6H, Ar–CH–(CH_3_)_2_); ^13^C-NMR (125 MHz, DMSO-d_6_, δ/ppm): 201.2 (C=S), 154.9 (C), 136.3 (C), 132.6 (C), 126.2 (CH), 122.5 (CH), 113.6 (CH), 74.4 (CH_2_), 25.9 (CH), 23.3 (2CH_3_), 21.4 (CH_3_); MS (ESI) *m*/*z*: [M + H]^+^ 224.0; anal. calculated for C_12_H_17_NOS (%): C, 64.54; H, 7.67; N, 6.27; S, 14.36; found (%): C, 64.76; H, 7.60; N, 6.35; S, 14.27.

#### 3.2.3. General Procedure for the Synthesis of 2-((2-isopropyl-5-methylphenoxy)methyl)-4-phenyl thiazole Derivatives **4a**–**e**

Equimolar quantities (1 mmol) of carbothioamide 3 and the corresponding α-haloketones were dissolved in dry acetone (3 mL) and stirred at room temperature for 6 h. The resulted precipitate was filtered under vacuum and washed with a solution of Na_2_CO_3_ until it was free of acid. The pure compounds were yielded through recrystallization from ethanol. 

*2-((2-isopropyl-5-methylphenoxy)methyl)-4-phenylthiazole* (**4a**): Yield 76%; m.p. 149–150 °C; FT-IR (KBr) ν_max_ cm^−1^: 3109 (C_5_–H thiazole str), 3049 (C-Har str), 2920 (C-Halif str), 1615 (C=N str), 1259 (C–O–C asym str), 1046 (C–O–C sym str); ^1^H-NMR (500 MHz, DMSO-d_6_, δ/ppm): 8.15 (s, 1H, thiazole-C_5_H), 7.98 (dd, *J* = 7.05; 1.25, 2H, Ar–H), 7.47 (t, 2H, Ar–H), 7.37 (t, 1H, Ar–H), 7.12 (d, *J* = 7.50, 1H), 6.97 (s, 1H, Ar–H), 6.79 (d, *J* = 7.50, 1H, Ar–H), 5.49 (s, 2H, O–CH_2_), 2.50-2.51 (m, 1H, Ar–CH–(CH_3_)_2_), 2.28 (s, 3H, Ar–CH_3_), 1.21 (d, *J* = 6.85, 6H, Ar–CH–(CH_3_)_2_); ^13^C-NMR (125 MHz, DMSO-d_6_, δ/ppm): 167.8 (C), 155.0 (C), 154.6 (C), 138.7 (C), 136.5 (C), 133.7 (C), 129.3 (2CH), 128.6 (CH), 126.4 (2CH), 126.3 (CH), 122.4 (CH), 115.2 (CH), 113.6 (CH), 67.5 (CH_2_), 26.8 (CH), 23.1 (2CH_3_), 21.4 (CH_3_); MS (ESI) *m*/*z*: [M + H]^+^ 324.3; anal. calculated for C_20_H_21_NOS (%): C, 74.27; H, 6.54; N, 4.33; S, 9.91; found (%): C, 74.40; H, 6.49; N, 4.26; S, 9.85. 

*4-(2-((2-isopropyl-5-methylphenoxy)methyl)thiazol-4-yl)benzonitrile* (**4b**): Yield 68%; m.p. 126–127 °C; FT-IR (KBr) ν_max_ cm^−1^: 3121 (C_5_-H thiazole str), 3031 (C-Har str), 2921 (C-Halif str), 2221 (C≡N str), 1616 (C=N str), 1256 (C–O–C asym str), 1048 (C–O–C sym str); ^1^H-NMR (500 MHz, DMSO-d_6_, δ/ppm): 8.43 (s, 1H, thiazole-C_5_H), 8.17 (d, *J* = 8.55 Hz, 2H, Ar–H), 7.93 (d, *J* = 8.55 Hz, 2H, Ar–H), 7.12 (d, *J* = 7.50, 1H, Ar–H), 6.95 (s, 1H, Ar–H), 6.79 (d, *J* = 7.50, 1H, Ar–H), 5.50 (s, 2H, O–CH_2_), 2.50-2.51 (m, 1H, Ar–CH– (CH_3_)_2_), 2.27 (s, 3H, Ar–CH_3_), 1.21 (d, *J* = 7.1 Hz, 6H, Ar–CH–(CH_3_)_2_); ^13^C-NMR (125 MHz, DMSO-d_6_, δ/ppm): 168.6 (C), 154.9 (C), 152.7 (C), 138.5 (C), 136.6 (C), 133.7 (C), 133.4 (2CH), 127.1 (2CH), 126.3 (CH), 122.5 (CH), 119.3 (C≡N), 118.6 (CH), 113.5 (CH), 110.8 (C), 67.4 (CH_2_), 26.8 (CH), 23.1 (2CH_3_), 21.4 (CH_3_); MS (ESI) *m*/*z*: [M+H]^+^ 349.3; anal. calculated for C_21_H_20_N_2_OS (%): C, 72.38; H, 5.79; N, 8.04; S, 9.20; found (%): C, 72.55; H, 5.71; N, 8.10; S, 9.29. 

*2-((2-isopropyl-5-methylphenoxy)methyl)-4-(4-nitrophenyl)thiazole* (**4c**): Yield 71%; m.p. 155–156 °C; FT-IR (KBr) ν_max_ cm^−1^: 3119 (C_5_-H thiazole str), 3042 (C-Har str), 2923 (C-Halif str), 1617 (C=N str), 1521 (N–O asym str), 1342 (N–O sym str), 1257 (C–O–C asym str), 1062 (C–O–C sym str); ^1^H-NMR (500 MHz, DMSO-d_6_, δ/ppm): 8.51 (s, 1H, thiazole-C_5_H), 8.67 (d, *J* = 8.5 Hz, 2H, Ar–H), 8.11 (d, *J* = 8.5 Hz, 2H, Ar–H), 7.12 (d, *J* = 7.50, 1H, Ar–H), 6.95 (s, 1H, Ar–H), 6.78 (d, *J* = 7.50, 1H, Ar–H), 5.51 (s, 2H, O–CH_2_), 2.50-2.51 (m, 1H, Ar–CH–(CH_3_)_2_), 2.27 (s, 3H, Ar–CH_3_), 1.20 (d, *J* = 6.50 Hz, 6H, Ar–CH–(CH_3_)2); ^13^C-NMR (125 MHz, DMSO-d_6_, δ/ppm): 168.6 (C), 154.9 (C), 152.8 (C), 145.2 (C), 138.5 (C), 137.0 (C), 134.0 (C), 133.3 (2CH), 127.3 (2CH), 126.3 (CH), 122.5 (CH), 118.7 (CH), 113.6 (CH), 67.4 (CH_2_), 26.8 (CH), 23.1 (2CH_3_), 21.4 (CH_3_); MS (ESI) *m*/*z*: [M+H]^+^ 369. 2; anal. calculated for C_20_H_20_N_2_O_3_S (%): C, 65.20; H, 5.47; N, 7.60; S, 8.70; found (%): C, 65.32; H, 5.54; N, 7.53; S, 8.61. 

*2-hydroxy-5-(2-((2-isopropyl-5-methylphenoxy)methyl)thiazol-4-yl)benzamide* (**4d**): Yield 77%; m.p. 197–198 °C; FT-IR (KBr) ν_max_ cm^−1^: 3415 (O–H str), 3264 (N–H str), 3180 (C_5_-H thiazole str), 3064 (C-Har str), 2920 (C-Halif str), 1671 (C=O str), 1617 (C=N str), 1255 (C-O-C asym str), 1097 (C–O–C sym str); ^1^H-NMR (500 MHz, DMSO-d_6_, δ/ppm): 10.03 (s, 1H, –OH), 9.63 (s, 1H, –NH_2_), 8.55 (s, 1H, –NH_2_), 8.47 (d, *J* = 1.95 Hz, 1H, Ar–H), 8.02 (dd, *J* = 8.55, 1.22 Hz, 1H, Ar–H), 7.98 (s, 1H, C_5_H thiazole), 7.12 (d, *J* = 7.50, 1H, Ar–H), 6.98 (d, *J* = 8.55 Hz, 2H, Ar–H), 6.94 (s, 1H, Ar–H), 6.78 (d, *J* = 7.50, 1H, Ar–H), 5.43 (s, 2H, O-CH_2_), 2.50–2.51 (m, 1H, Ar–CH–(CH_3_)_2_), 2.27 (s, 3H, Ar–CH_3_), 1.20 (d, *J* = 7.05 Hz, 6H, Ar–CH–(CH_3_)2); ^13^C-NMR (125 MHz, DMSO-d_6_, δ/ppm): 172.2 (C), 167.7 (C), 161.2 (C), 155.0 (C), 154.1 (C), 136.5 (C), 133.7 (C), 132.2 (CH), 126.3 (2CH), 125.5 (C), 122.4 (CH), 118.2 (CH), 115.2 (C), 113.5 (CH), 113.3 (CH), 67.5 (CH_2_), 26.8 (CH), 23.1 (2CH_3_), 21.4 (CH_3_); MS (ESI) *m*/*z*: [M+H]^+^ 383.4; anal. calculated for C_21_H_22_N_2_O_3_S (%): C, 65.95; H, 5.80; N, 7.32; S, 8.38; found (%): C, 66.07; H, 5.71; N, 7.29; S, 8.47.

*2-((2-isopropyl-5-methylphenoxy)methyl)-4-(p-tolyl)thiazole* (**4e**): Yield 78%; m.p. 194–195 °C; FT-IR (KBr) ν_max_ cm^−1^: 3117 (C_5_-H thiazole str), 3071 (C-Har str), 2923 (C-Halif str), 1616 (C=N str), 1257 (C–O–C asym str), 1097 (C–O–C sym str); ^1^H-NMR (500 MHz, DMSO-d_6_, δ/ppm): 8.07 (s, 1H, thiazole-C_5_H), 7.98 (d, *J* = 8.5 Hz, 2H, Ar–H), 7.31 (d, *J* = 8.5 Hz, 2H, Ar–H), 7.13 (d, *J* = 7.50, 1H, Ar–H), 6.94 (s, 1H, Ar–H), 6.80 (d, *J* = 7.50, 1H, Ar–H), 5.49 (s, 2H, O–CH_2_), 2.50–2.51 (m, 1H, Ar–CH–(CH_3_)_2_), 2.37 (s, 3H, Ar–CH_3_), 2.29 (s, 3H, Ar–CH_3_), 1.20 (d, *J* = 6.5 Hz, 6H, Ar–CH–(CH_3_)_2_); ^13^C-NMR (125 MHz, DMSO-d_6_, δ/ppm): 168.8 (C), 155.0 (C), 153.1 (C), 138.1 (C), 137.1 (C), 136.8 (C), 131.2 (C), 133.8 (2CH), 127.4 (2CH), 127.1 (CH), 123.1 (CH), 115.9 (CH), 113.8 (CH), 67.4 (CH_2_), 26.8 (CH), 23.1 (2CH_3_), 21.4 (CH_3_), 21.1 (CH_3_); MS (ESI) *m*/*z*: [M + H]^+^ 338.4; anal. calculated for C_21_H_23_NOS (%): C, 74.74; H, 6.87; N, 4.15; S, 9.50; found (%): C, 74.91; H, 6.72; N, 4.23; S, 9.46.

#### 3.2.4. Synthesis of 2-(2-(2-isopropyl-5-methylphenoxy)-1-phenylethylidene)hydrazine-1-carbo-thioamide **6**

To a solution containing thymol (10 mmol) and potassium hydroxide (KOH) (11 mmol) in dry acetone (10 mL) cooled at 0 °C in an ice–salt bath, equimolar quantities of 2-Br-acetophenone (10 mmol dissolved in 2 mL of dry acetone) was added dropwise under stirring, over a period of 1h. The reaction mixture was further refluxed on a steam bath, at 55–60 °C, for 8 h, until TLC indicated the completion of the chemical process. The solvent was removed under reduced pressure, using a rotary evaporator, thus obtaining the intermediate ketone **5**. Then, equimolar quantities of thiosemicarbazide were added, and the reaction mixture was refluxed in absolute ethanol (15 mL) for 12 h, in the presence of catalytic amounts of glacial acetic acid (2 drops). After cooling at room temperature overnight, the resulting precipitate was filtered under vacuum, dried, and recrystallized from ethanol to yield the pure intermediate **6**. 

Yield 67%; m.p. 163.5 °C; FT-IR (KBr) ν_max_ cm^−1^: 3237, 3154 (N–H str), 3057 (C-Har str), 2922 (C-Halif str), 1613 (C=N str), 1249 (C–O–C asym str), 1161 (C=S str), 1063 (C–O–C sym str); ^1^H-NMR (500 MHz, DMSO-d_6_, δ/ppm): 10.80 (s, 1H, NH), 8.45 (s, 1H, NH_2_), 8.07 (s, 1H, NH_2_), 7.93 (d, *J* = 2 Hz, 1H, Ar–H), 7.92 (t, 1H, Ar–H), 7.39 (d, *J* = 2 Hz, 1H, Ar–H), 7.38 (t, 2H, Ar–H), 7.05 (d, *J* = 7.50, 1H, Ar–H), 6.88 (s, 1H, Ar–H), 6.76 (d, *J* = 7.50, 1H, Ar–H), 5.30 (s, 2H, O–CH_2_), 2.50-2.51 (m, 1H, Ar–CH–(CH_3_)_2_), 2.28 (s, 3H, Ar–CH_3_), 1.01 (d, *J* = 7 Hz, 6H, Ar–CH–(CH_3_)_2_); ^13^C-NMR (125 MHz, DMSO-d_6_, δ/ppm): 179.6 (C=S), 145.3 (C=N), 136.2 (C), 136.1 (C), 133.7 (C), 129.6 (CH), 128.0 (2CH), 127.6 (CH), 126.1 (2CH), 122.1 (CH), 113.2 (CH), 61.7 (CH_2_), 26.2 (CH), 22.9 (2CH_3_), 21.4 (CH_3_); MS (ESI) *m*/*z*: [M + H]^+^ 342.2; anal. calculated for C_19_H_23_N_3_OS (%): C, 66.83; H, 6.79; N, 12.31; S, 9.39; found (%): C, 66.98; H, 6.68; N, 12.27; S, 9.31.

#### 3.2.5. General Procedure for the Synthesis of 2-(2-(2-(2-isopropyl-5-methylphenoxy)-1-phenyl ethylidene)hydrazineyl)-4-phenylthiazole Derivatives **7a**–**e**

To a solution of hydrazinyl-1-carbothioamide **6** (1 mmol) dissolved in dry acetone (3 mL), equimolar quantities of the corresponding α-haloketones were added, and the mixture was stirred at room temperature for 6 h. The resulted precipitate was filtered under vacuum and washed with a solution of Na_2_CO_3_ until free of acid. The pure compounds were yielded through recrystallization from ethanol. 

*2-(2-(2-(2-isopropyl-5-methylphenoxy)-1-phenylethylidene)hydrazineyl)-4-phenylthiazole* (**7a**): Yield 76%; m.p. 174–175 °C; FT-IR (KBr) ν_max_ cm^−1^: 3230 (N–H str), 3122 (C_5_–H thiazole str), 3031 (C-Har str), 2927 (C-Halif str), 1630 (C=N str), 1248 (C–O–C asym str), 1068 (C–O–C sym str); ^1^H-NMR (500 MHz, DMSO-d_6_, δ/ppm): 8.29 (s, 1H, NH), 7.99 (d, *J* = 6.00 Hz, 2H, Ar–H), 7.81 (d, *J* = 7.2 Hz, 2H, Ar–H), 7.71 (s, 1H, thiazole-C_5_H), 7.48 (t, 2H, Ar–H), 7.36 (t, 1H, Ar–H), 7.31 (t, 2H, Ar–H), 7.25 (t, 1H, Ar–H), 7.03 (d, *J* = 7.50, 1H, Ar–H), 6.93 (s, 1H, Ar–H), 6.77 (d, *J* = 7.50, 1H, Ar–H), 5.28 (s, 2H, O–CH_2_), 2.50-2.51 (m, 1H, Ar–CH–(CH_3_)_2_), 2.28 (s, 3H, Ar–CH_3_), 1.01 (d, *J* = 7.1 Hz, 6H, Ar–CH–(CH_3_)2); ^13^C-NMR (125 MHz, DMSO-d_6_, δ/ppm): 163.2 (C), 155.0 (C), 146.1 (C=N), 144.0 (C), 137.0 (C), 136.8 (C), 134.2 (C), 133.7 (C), 129.8 (2CH), 129.2 (CH), 128.3 (2CH), 127.0 (2CH), 127.2 (CH), 127.4 (CH), 125.5 (2CH), 123.1 (CH), 113.9 (CH), 110.3 (CH), 65.8 (CH_2_), 26.3 (CH), 23.1 (2CH_3_), 21.5 (CH_3_); MS (ESI) *m*/*z*: [M + H]^+^ 442.6; anal. calculated for C_27_H_27_N_3_OS (%): C, 73.44; H, 6.16; N, 9.52; S, 7.26; found (%): C, 73.78; H, 6.09; N, 9.61; S, 7.35. 

*4-(2-(2-(2-(2-isopropyl-5-methylphenoxy)-1-phenylethylidene)hydrazineyl)thiazol-4-yl)benzonitrile* (**7b**): Yield 78%; m.p. 192–194 °C; FT-IR (KBr) ν_max_ cm^−1^: 3246 (N-H str), 3130 (C_5_-H thiazole str), 3052 (C-Har str), 2921 (C-Halif str), 2310 (C≡N str), 1607 (C=N str), 1241 (C–O–C asym str), 1078 (C–O–C sym str); ^1^H-NMR (500 MHz, DMSO-d_6_, δ/ppm): 8.34 (s, 1H, NH), 8.05 (d, *J* = 8.3 Hz, 2H, Ar–H), 7.87 (d, *J* = 8.3 Hz, 2H, Ar–H), 7.76 (d, *J* = 8.3 Hz, 2H, Ar–H), 7.67 (s, 1H, thiazole-C_5_H), 7.42 (t, 1H, Ar–H), 7.37 (t, 2H, Ar–H), 7.05 (d, *J* = 7.50, 1H, Ar–H), 6.92 (s, 1H, Ar–H), 6.75 (d, *J* = 7.50, 1H, Ar–H), 5.29 (s, 2H, O–CH_2_), 2.50-2.51 (m, 1H, Ar–CH–(CH_3_)_2_), 2.28 (s, 3H, Ar–CH_3_), 1.00 (d, *J* = 7.1 Hz, 6H, Ar–CH–(CH3)2); ^13^C-NMR (125 MHz, DMSO-d_6_, δ/ppm): 162.8 (C), 155.1 (C), 148.2 (C=N), 143.5 (C), 136.6 (C), 136.2 (C), 136.1 (C), 133.6 (C), 133.1 (C), 129.1 (2CH), 128.7 (CH), 128.7 (2CH), 126.7 (2CH), 126.5 (2CH), 126.1 (CH), 121.8 (CH), 119.4 (C≡N), 113.1 (CH), 110.0 (CH), 61.2 (CH_2_), 26.3 (CH), 23.0 (2CH_3_), 21.5 (CH_3_); MS (ESI) *m*/*z*: [M + H]^+^ 467.5; anal. calculated for C_28_H_26_N_4_OS (%): C, 72.08; H, 5.62; N, 12.01; S, 6.87; found (%): C, 72.21; H, 5.65; N, 12.04; S, 6.93.

*2-(2-(2-(2-isopropyl-5-methylphenoxy)-1-phenylethylidene)hydrazineyl)-4-(4-nitrophenyl)thiazole* (**7c**): Yield 75%; m.p. 168–169 °C; FT-IR (KBr) ν_max_ cm^−1^: 3243 (N–H str), 3100 (C_5_-H thiazole str), 3042 (C-Har str), 2921 (C-Halif str), 1617 (C=N str), 1510 (N–O asym str), 1341 (N–O sym str), 1242 (C–O–C asym str), 1036 (C–O–C sym str); ^1^H-NMR (500 MHz, DMSO-d_6_, δ/ppm): 8.42 (s, 1H, NH), 8.21 (d, *J* = 7.5 Hz, 2H, Ar–H), 7.92 (d, *J* = 7.5 Hz, 2H, Ar–H), 7.58 (d, *J* = 7.10 Hz, 2H, Ar–H), 7.62 (s, 1H, thiazole-C_5_H), 7.38 (t, 1H, Ar–H), 7.33 (t, 2H, Ar–H), 7.12 (d, *J* = 7.50, 1H, Ar–H), 6.94 (s, 1H, Ar–H), 6.77 (d, *J* = 7.50, 1H, Ar–H), 5.27 (s, 2H, O–CH_2_), 2.50-2.51 (m, 1H, Ar–CH–(CH_3_)_2_), 2.29 (s, 3H, Ar–CH_3_), 1.20 (d, *J* = 7.2 Hz, 6H, Ar–CH–(CH_3_)_2_); ^13^C-NMR (125 MHz, DMSO-d_6_, δ/ppm): 163.7 (C), 156.2 (C), 149.2 (C–NO2), 147.5 (C=N), 143.8 (C), 138.0 (C), 137.1 (C), 136.5 (C), 133.6 (C), 129.2 (CH), 128.5 (2CH), 127.5 (CH), 127.1 (2CH), 126.0 (2CH), 125.6 (2CH), 123.0 (CH), 114.7 (CH), 110.4 (CH), 62.7 (CH_2_), 26.4 (CH), 22.9 (2CH_3_), 21.3 (CH_3_); MS (ESI) *m*/*z*: [M + H]^+^ 487.4; anal. calculated for C_27_H_26_N_4_O_3_S (%): C, 66.65; H, 5.39; N, 11.51; S, 6.59; found (%):C, 66.82; H, 5.27; N, 11.58; S, 6.64.

*2-hydroxy-5-(2-(2-(2-(2-isopropyl-5-methylphenoxy)-1-phenylethylidene)hydrazineyl)thiazol-4-yl)benzamide* (**7d**): Yield 80%; m.p. 197–198 °C; FT-IR (KBr) ν_max_ cm^−1^: 3474 (O–H str), 3409, 3232 (N–H str), 3123 (C_5_–H thiazole str), 3042 (C-Har str), 2918 (C-Halif str), 1654 (C=O str), 1616 (C=N str), 1246 (C–O–C asym str), 1091 (C–O–C sym str); ^1^H-NMR (500 MHz, DMSO-d_6_, δ/ppm): 13.30 (s, 1H, –OH), 8.54 (s, 1H, NH), 8.43 (s, 1H, NH_2_), 7.94 (s, 1H, NH_2_), 8.47 (d, *J* = 2.50 Hz, 1H, Ar–H), 8.02 (dd, *J* = 8.55, 2.22 Hz, 1H, Ar–H), 7.76 (s, 1H, thiazole–C_5_H), 7.77 (d, *J* = 1.5 Hz, 1H, Ar–H), 7.48 (d, *J* = 1.5 Hz, 1H, Ar–H), 7.41 (t, 1H, Ar–H), 7.37 (t, 2H, Ar–H), 7.12 (d, *J* = 8.00, 1H, Ar–H), 6.98 (d, *J* = 8.5 Hz, 2H, Ar–H), 6.95 (s, 1H, Ar–H), 6.79 (d, *J* = 8.00, 1H, Ar–H), 5.47 (s, 2H, O–CH_2_), 2.50-2.51 (m, 1H, Ar–CH–(CH_3_)_2_), 2.27 (s, 3H, Ar–CH_3_), 1.20 (d, *J* = 6.85 Hz, 6H, Ar–CH–(CH_3_)_2_); ^13^C-NMR (125 MHz, DMSO-d_6_, δ/ppm): 172.2 (C=O), 167.7 (C), 161.2 (C), 155.2 (C), 145.8 (C=N), 141.3 (C), 136.2 (C), 136.6 (C), 133.2 (C), 133.7 (C), 131.7 (CH), 129.1 (CH), 128.7 (2CH), 126.7 (CH), 126.0 (CH), 125.5 (2CH), 121.8 (CH), 117.9 (CH), 115.2 (CH), 113.4 (C), 113.1 (CH), 67.5 (CH_2_), 26.8 (CH), 23.1 (2CH_3_), 21.4 (CH_3_); MS (ESI) *m*/*z*: [M + H]^+^ 501.4; anal. calculated for C_28_H_28_N_4_O_3_S (%): C, 67.18; H, 5.64; N, 11.19; S, 6.40; found (%): C, 67.35; H, 5.59; N, 11.25; S, 6.51.

*2-(2-(2-(2-isopropyl-5-methylphenoxy)-1-phenylethylidene)hydrazineyl)-4-(p-tolyl)thiazole* (**7e**): Yield 78%; m.p. 158.5–159.5 °C; FT-IR (KBr) ν_max_ cm^−1^: 3236 (N–H str), 3144 (C_5_–H thiazole str), 3030 (C-Har str), 2922 (C-Halif str), 1616 (C=N str), 1242 (C–O–C asym str), 1078 (C–O–C sym str); ^1^H-NMR (500 MHz, DMSO-d6, δ/ppm): 8.19 (s, 1H, NH), 7.98 (s, 1H, thiazole–C_5_H), 7.91 (d, *J* = 9.00 Hz, 2H, Ar–H), 7.82 (d, *J* = 7.5 Hz, 2H, Ar–H), 7.45 (d, *J* = 7.5 Hz, 2H, Ar–H), 7.39 (t, 2H, Ar–H), 7.35 (t, 1H, Ar–H), 7.12 (d, *J* = 7.50, 1H, Ar–H), 6.93 (s, 1H, Ar–H), 6.81 (d, *J* = 7.50, 1H, Ar–H), 5.35 (s, 2H, O–CH_2_), 2.50-2.51 (m, 1H, Ar–CH–(CH_3_)_2_), 2.36 (s, 3H, Ar–CH_3_), 2.29 (s, 3H, Ar–CH_3_), 1.20 (d, *J* = 7.1 Hz, 6H, Ar–CH–(CH_3_)_2_); ^13^C-NMR (125 MHz, DMSO-d_6_, δ/ppm): 163.1 (C), 155.1 (C), 145.5 (C=N), 143.8 (C), 138.5 (C), 137.1 (C), 136.9 (C), 135.0 (C), 133.7 (2CH), 133.6 (C), 129.0 (CH), 128.4 (2CH), 127.5 (2CH), 127.1 (CH), 125.9 (2CH), 123.0 (CH), 114.0 (CH), 110.3 (CH), 67.1 (CH_2_), 26.7 (CH), 23.0 (2CH_3_), 21.4 (CH_3_), 21.1 (CH_3_); MS (ESI) *m*/*z*: [M + H]^+^ 456.5; anal. calculated for C_28_H_29_N_3_OS (%): C, 73.81; H, 6.42; N, 9.22; S, 7.04; found (%): C, 73.65; H, 6.35; N, 9.31; S, 7.12.

### 3.3. Anti-Candida Activity Assay

The in vitro anti-*Candida* screening was done according to the guidelines of the Clinical Laboratory Standards Institute (CLSI) [45]. The broth microdilution method was employed for the determination of MIC and MFC, as previously reported [18]. All the tested fungal strains were obtained from the Food Biotechnology Laboratory, Life Sciences Institute, University of Agricultural Sciences and Veterinary Medicine Cluj-Napoca, Romania. 

### 3.4. Molecular Docking

The binding affinity of the newly synthesized compounds (**4a**–**e**, **7a**–**e**) to the catalytic site of the target fungal lanosterol C14α-demethylase (CYP51) was evaluated through a molecular docking study, using AutoDock 4.2.6 [46].

The docking protocol in terms of search space size, grid spacing, center Cartesian coordinates, and 2Å cluster analysis was applied as previously reported [47,48], with the exception of the number of generated conformations for each ligand. This parameter was increased to 100 in order to assess a higher reproducibility of the results obtained from the molecular docking study [18]. 

The inhibition constant (Ki) values were calculated based on the in silico predicted binding (∆G), using the following formula: Ki = e^((∆G × 1000)/(R × T)) (R represents the Regnault constant = 198,719 kcal/(K × mol) and T = 298.15 K). 

Visualization and analysis of the docking results were performed using UCSF Chimera 1.10.2 [49].

### 3.5. ADMET Predictions

A SwissADME web tool [50] was employed for the in silico assignment of drug-likeness for the newly synthesized compounds. 

The pharmacokinetic profile (ADME) and drug-likeness of the tested compounds was assessed through the analysis of a series of physicochemical properties, according to the Lipinski Rule of Five [23]: molecular weight (MW), hydrogen bond acceptors (HBA), hydrogen bond donors (HBD), and Moriguchi LogP (mLogP). Additional molecular property-related filters for oral bioavailability, as topological polar surface area (tPSA) and the number of rotatable bonds (RoB), were also applied [51]. 

Oral bioavailability in terms of human gastrointestinal absorption and blood–brain barrier (BBB) permeation of the target compounds were evaluated based on tPSA calculated values and their predicted ability to act as glycoprotein P (Pgp) substrates. The percentage of gastrointestinal absorption (%GI Abs) was calculated using the formula: %GI Abs = 109 − (0.345 × tPSA) [27]. 

The predicted potential for the cross-inhibition of human CYP450 isoenzymes was used to assess the susceptibility for pharmacokinetic drug–drug interactions of the newly synthesized molecules.

### 3.6. Protein-Binding Study

BSA fraction V was purchased from Merck (Darmstadt, Germany) and used as supplied, without further purification. All the other chemicals were of analytical grade purity.

All the fluorescence emission spectra were recorded on a Jasco FP-6500 spectrofluorometer equipped with a DC-powered 150 W Xenon lamp and a 1.0-cm quartz cell. Spectral analysis was performed at room temperature under simulated physiological conditions (pH = 7.4) by excitation of the probes at 289 nm and an emission scan ranging from 300 to 500 nm. Excitation and emission slit width was fixed at 3 nm. 

It is worthwhile to highlight that the BSA fluorescence intensity is reduced by the UV-VIS absorption of the ligands at the excitation and emission wavelengths [14]. For the tested compounds, the inner-filter effect of fluorescence was ignored, as they have no absorbance at the above-mentioned parameters, at the used concentrations.

BSA stock solution was prepared in a Tris-HCl buffer solution (consisted of Tris 0.05 M and adjusted to pH = 7.4 using HCl 36%) containing NaCl 0.05 M, and was kept in the dark at 4 °C. The stock solutions of the tested compounds (15 mM) were prepared in DMSO and accordingly diluted to obtain a final concentration ranging from 0.6 to 1.8 μM. 

The probes were prepared by mixing a fixed concentration of BSA solution (1.5 μM) with appropriate amounts of the tested compound **7e** in a 10-mL volumetric flask and diluting with Tris buffer solution to obtain final ligand concentrations of 0.3, 0.6, 0.9, 1.2, 1.5 and 1.8 μM. 

## 4. Conclusions

Two series of novel 1,3-thiazole derivatives were designed as potential anti-*Candida* agents and synthesized through a Hantzsch condensation protocol. The chemical structures were confirmed by spectral analysis. 

In order to evaluate the drug-likeness and pharmacokinetic profile of the newly synthesized compounds, an in silico ADMET screening was performed. The obtained results support their suitability for further development as orally active drugs. Additionally, the distribution profile of the most biologically active compound (**7e**), which influences the access to the target site in vivo, was investigated through a serum albumin-binding study, using fluorescence spectroscopy. The experiment showed a strong binding interaction of the tested compound with the target carrier protein, mediated by the formation of a ground-state nonfluorescent complex.

The antifungal potential of the novel 1,3-thiazoles, in terms of MIC and MFC, was evaluated in vitro against three pathogenic Candida strains. The results of the anti-*Candida* assay pointed to some structure-dependent differences in the inhibitory activity, which were related to the C_2_-substitution of the thiazole ring and para-phenyl substituent properties. Compounds **7a** and **7e** are the most promising ones, exhibiting substantially lower MIC values (7.81 μg/mL and 3.9 μg/mL) as compared with the reference drug fluconazole (15.62 μg/mL). 

A molecular docking study was performed toward fungal lanosterol-C14α-demethylase (CYP51), aiming to investigate the molecular mechanism of action of the newly synthesized compounds. All the tested molecules demonstrated a good binding affinity toward the target enzyme. The induced enzyme inhibition is not related to the covalent coordination of the heme Fe^2+^, as in the case of classical antifungal azoles, but they compete with the physiological substrate of the CYP51 for the access channel to the active site.

The above-mentioned data represent a helpful support for the rational design and development of novel antifungal drugs with improved activity and pharmacokinetic properties, and lower toxicity.

## Figures and Tables

**Table 1:** The minimum inhibitory concentration values (MIC, μg/mL) of the newly synthesized 4-phenyl-1,3-thiazole (**4a**–**e**) and 2-hydrazinyl-4-phenyl-1,3-thiazole (**7a**–**e**) derivatives.

Compound	*C. albicans* ATCC 10231	*C. parapsilosis* ATCC 22019	*C. zeylanoides* ATCC 201082
**4a**	62.5	62.5	125
**4b**	62.5	62.5	125
**4c**	62.5	62.5	125
**4d**	62.5	62.5	125
**4e**	62.5	62.5	125
**7a**	7.81	15.62	62.5
**7b**	62.5	62.5	62.5
**7c**	62.5	62.5	125
**7d**	62.5	62.5	125
**7e**	3.9	15.62	15.62
Fluconazole	15.62	7.81	15.62

**Table 2:** The minimum fungicidal concentration values (MFC, μg/mL) of the newly synthesized 4-phenyl-1,3-thiazole (**4a**–**e**) and 2-hydrazinyl-4-phenyl-1,3-thiazole (**7a**–**e**) derivatives.

Compound	*C. albicans* ATCC 10231	*C. parapsilosis* ATCC 22019	*C. zeylanoides* ATCC 201082
**4a**	125	125	250
**4b**	125	125	250
**4c**	125	125	250
**4d**	125	125	250
**4e**	125	125	250
**7a**	15.62	31.24	125
**7b**	125	125	125
**7c**	125	125	250
**7d**	125	125	250
**7e**	7.8	31.24	31.24
Fluconazole	31.24	15.62	31.24

**Table 3:** The predicted binding interaction energy (ΔG, kcal/mol), the consequent inhibition constant (Ki, nM) and the cluster analysis containing the best conformation of each compound given by AutoDs.

Compound	Best Binding Conformation		The 2 Å Cluster Containing the Top Binding Conformation				Number of Distinct Clusters	
	ΔG (kcal/mol)	Ki (nM)	NoC ^1^	Average ΔG (kcal/mol)	Standard Deviation			
					ΔG (kcal/mol)	Cartesian Coordinates	Total	Multi-Member
**4a**	−10.51	19.77	19	−9.89	0.54	0.73	26	15
**4b**	−11.49	3.78	41	−10.60	0.46	0.36	21	15
**4c**	−10.93	9.73	32	−9.95	0.56	0.51	23	12
**4d**	−10.83	11.52	6	−10.52	0.27	0.76	38	19
**4e**	−11.00	8.65	27	−10.63	0.51	0.47	23	16
**7a**	−12.52	0.66	3	−11.35	1.20	1.04	50	18
**7b**	−13.21	0.21	14	−12.51	0.74	0.50	42	16
**7c**	−12.20	1.14	1	–	–	–	44	18
**7d**	−12.39	0.83	1	–	–	–	64	17
**7e**	−12.71	0.48	10	−12.63	0.70	0.70	48	19

^1^ = number of conformations.

**Table 4:** Computationally predicted drug-like descriptors of compounds **4a**–**e** and **7a**–**e**.

Compound	MW ^1^ (Da)	RoB ^2^	HBA ^3^	HBD ^4^	tPSA ^5^	mLogP ^6^	Lipinski Violations	Veber Violations
**4a**	323.45	5	2	0	50.36	3.54	0	0
**4b**	348.46	5	3	0	74.15	2.81	0	0
**4c**	368.45	6	4	0	96.18	2.39	0	0
**4d**	382.48	6	4	2	113.68	2.07	0	0
**4e**	337.48	5	2	0	50.36	3.76	0	0
**7a**	441.59	8	3	1	74.75	4.49	1	0
**7b**	466.60	8	4	1	98.54	3.78	0	0
**7c**	486.59	9	5	1	120.57	3.46	0	0
**7d**	500.61	9	5	3	138.07	3.11	1	0
**7e**	455.61	8	3	1	74.75	4.68	1	0

^1^ Molecular weight; ^2^ Number of rotatable bonds; ^3^ Number of hydrogen bond acceptors; ^4^ Number of hydrogen bond donors; ^5^ Topological polar surface area; ^6^ Moriguchi logarithm of the compound partition coefficient between n-octanol and water.

**Table 5:** Computationally predicted pharmacokinetic profile of compounds **4a**–**e** and **7a**–**e**.

Compound	%GI Abs ^1^	BBBP ^2^	PGP ^3^ Substrate	CYP450 Inhibition	
				CYP3A4	CYP2C9
**4a**	**91.62**	**Yes**	Yes	Yes	Yes
**4b**	83.41	No	Yes	Yes	Yes
**4c**	75.81	No	No	Yes	Yes
**4d**	69.78	No	No	Yes	Yes
**4e**	91.62	No	Yes	Yes	Yes
**7a**	83.21	No	Yes	Yes	No
**7b**	75.00	No	Yes	Yes	Yes
**7c**	67.40	No	No	Yes	No
**7d**	61.36	No	No	No	No
**7e**	83.21	No	Yes	Yes	No

^1^ gastrointestinal absorption (%); ^2^ blood–brain barrier permeability; ^3^ P-glycoprotein.

**Table 6:** Stern–Volmer equation constants for the interaction of BSA with the compound **7e** at room temperature (λmax BSA = 341 nm).

Compound	K_q_ × 10^13^ (L/mol·s)	^a^ K_SV_ × 10^5^ (L/mol)	^b^ R^2^
**7e**	3.29 ± 0.0017	1.97± 0.0124	0.9856

^a^ K_SV_ = K_q_ × τ_0,_ τ_0 ≈_ 6 ns; ^b^ R^2^ is the correlation coefficient.

**Table 7:** Binding constant and binding sites for compound **7e** binding into BSA.

Compound	n	logK_b_	K_b_ × 10^5^ (M^−1^)	R^2^
**7e**	0.873 ± 0.0355	−0.6448 ± 0.0094	2.26	0.9917
